# Development of an auto-inducible expression system by nitrogen sources switching based on the nitrogen catabolite repression regulation

**DOI:** 10.1186/s12934-022-01794-5

**Published:** 2022-04-28

**Authors:** Qin Yan, Laichuang Han, Xinyue Liu, Cuiping You, Shengmin Zhou, Zhemin Zhou

**Affiliations:** 1 Key Laboratory of Industrial Biotechnology (Ministry of Education), School of Biotechnology, Jiangnan University, Wuxi, 214122 Jiangsu China; 2 State Key Laboratory of Bioreactor Engineering, School of Biotechnology, East China University of Science and Technology, Shanghai, China

**Keywords:** Gene expression system, Auto-inducible, Nitrogen catabolite repression, *Aspergillus nidulans*

## Abstract

**Background:**

The construction of protein expression systems is mainly focused on carbon catabolite repression and quorum-sensing systems. However, each of these regulatory modes has an inherent flaw, which is difficult to overcome. Organisms also prioritize using different nitrogen sources, which is called nitrogen catabolite repression. To date, few gene regulatory systems based on nitrogen catabolite repression have been reported.

**Results:**

In this study, we constructed a nitrogen switching auto-inducible expression system (NSAES) based on nitrogen catabolite regulation and nitrogen utilization in *Aspergillus nidulans*. The P_niaD_ promoter that is highly induced by nitrate and inhibition by ammonia was used as the promoter. Glucuronidase was the reporter protein. Glucuronidase expression occurred after ammonium was consumed in an ammonium and nitrate compounding medium, achieving stage auto-switching for cell growth and gene expression. This system maintained a balance between cell growth and protein production to maximize stress products. Expressions of glycosylated and secretory proteins were successfully achieved using this auto-inducible system.

**Conclusions:**

We described an efficient auto-inducible protein expression system based on nitrogen catabolite regulation. The system could be useful for protein production in the laboratory and industrial applications. Simultaneously, NSAES provides a new auto-inducible expression regulation mode for other filamentous fungi.

**Supplementary information:**

The online version contains supplementary material available at 10.1186/s12934-022-01794-5.

## Background

Auto-inducible regulation of gene expression, which can automatically switch from growth to production mode for balancing biomass and product synthesis, is increasingly used in protein expression, metabolic engineering, and synthetic biology [1–3]. However, the development of auto-inducible regulatory modules mainly relies on regulators involved in carbon catabolite repression (CCR) and quorum-sensing systems [4]. Each regulatory mode has an inherent flaw that is difficult to overcome. For CCR-based systems, some types of carbon sources seriously affect the physical properties of the medium, such as the detriment of viscosity to high-density fermentation. However, some carbon sources, such as isopropyl β-d-1-thiogalactopyranoside (IPTG), are expensive. For auto-inducible regulation based on a quorum-sensing system, ultra-sensitivity makes it difficult to fine-tune the timing and strength of gene expression [5, 6]. In addition, only some microbial quorum-sensing systems have been studied and applied to construct artificial genetic circuits [7, 8]. More importantly, quorum-sensing systems are often associated with microbial pathogenicity, and the safety of their application must be carefully considered [9, 10].

Similar to CCR, organisms have different priorities for using different nitrogen sources (nitrogen catabolite repression). Organisms prefer multiple nitrogen sources, such as ammonia, glutamine, and glutamate [11], which provide diverse options for constructing gene regulatory systems based on NCR. Nitrate is also an excellent nitrogen source for filamentous fungi; however, it is not utilized in the presence of preferred nitrogen. Previous studies have shown that nitrate decreases protease secretion but increases the production of other enzymes because of its elimination to the NCR [12, 13]. However, few gene regulatory systems based on NCR have been reported.

Filamentous fungi, with a powerful ability to secrete proteins and protein glycosylation system similar to those of higher organisms, are considered advantageous cell factories for producing enzymes, secondary metabolites, and drugs [14–17]. Among them, *Aspergillus* species are attractive candidates for expressing genes of interest owing to their well-characterized physiologies, morphologies, genetic backgrounds, and their ease-of-use [18]. In particular, *Aspergillus nidulans* grow rapidly on inexpensive media, shortening the fermentation period and reducing production costs. Therefore, *A. nidulans* has the potential to be an ideal chassis cell for various industrial applications.

**Fig. 1:**
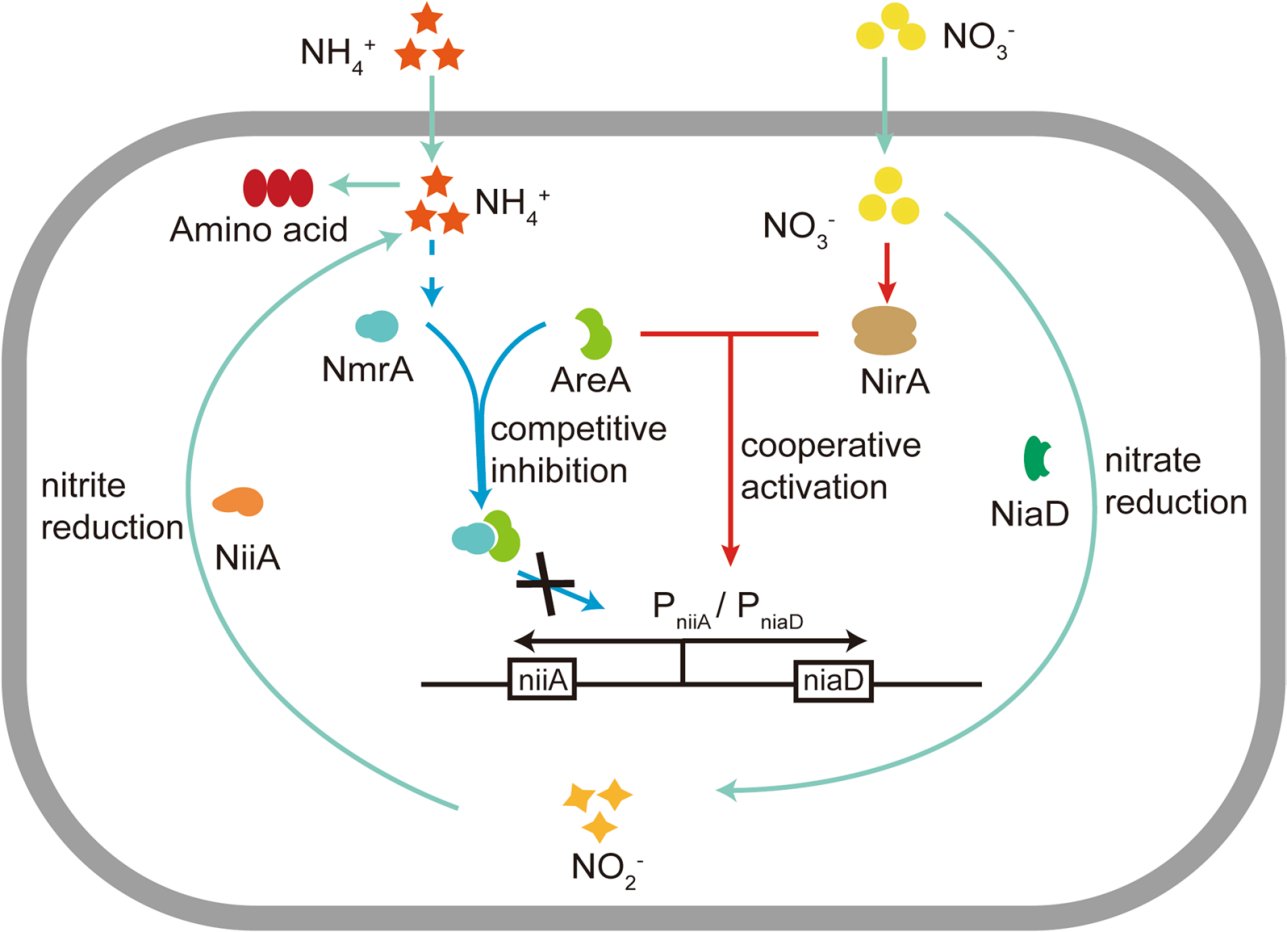
Metabolic regulation of NO_3_^−^ and NH_4_^+^ in *A.* *nidulans. *The expressions of *niiA* and *niaD* are activated by the cooperation of transcription factors NirA and AreA (red arrow), while this process is repressed by NH_4_^+^ activated NmrA and P_niiA_/P_niaD_ compete for AreA (blue arrow)

Nitrate assimilation is a crucial process in nitrogen acquisition in *A. nidulans. A. nidulans* possesses enzymatic and regulatory systems that allow the conversion of nitrate to nitrite and further to ammonium, which is then incorporated into amino acids and other metabolites [19]. Nitrate is converted to ammonia by nitrate reductase and nitrite reductase. However, this process is inhibited by ammonia because ammonia is the preferred nitrogen source for *A. nidulans* rather than nitrate [20]. The genetics of the fungal nitrate assimilation pathway have been extensively investigated. As shown in Fig. 1, the expression of nitrate and nitrite reductases (NiiA and NiaD, respectively) in *A. nidulans* is controlled by bidirectional promoter P_niiA_/P_niaD_ with a length of 1267 bp (Additional file 1: Fig. S3). The promoter including ten AreA binding sites and four NirA binding sites and it is synergistically activated by the transcription factors NirA and AreA [21, 22]. In the presence of NH_4_^+^, another transcription factor, NmrA, is activated and binds to AreA. The binding represses the cooperation between NirA and AreA [23, 24]. Without the cooperation of AreA, P_niiA_/P_niaD_ promoter cannot be activated by NirA, resulting in the inhibition of nitrate metabolism. AreA is released from NmrA only after NH_4_^+^ exhaustion. The released AreA cooperates with NirA to activate P_niiA_/P_niaD_ promoter. Owing to its vital role in nitrogen source utilization, P_niiA_/P_niaD_ the ideal promoter to construct an NCR-based auto-inducible expression system in *A. nidulans*.

Here, we developed a nitrogen-switching auto-inducible expression system (NSAES) in *A. nidulans* based on the NCR regulation. P_niaD_, with strong activity and tight regulation, was used as the promoter, and glucuronidase was employed as the reporter gene for NSAES construction. P_niaD_ was activated by nitrate for glucuronidase expression after ammonium was removed in an ammonium and nitrate compounding medium, achieving a stage separation function for cell growth and gene expression and nitrogen-switching auto-inducible expression. The conditional lethal gene *pyrG* was employed to confirm that NSAES could maintain a balance between cell growth and stress protein production. Expressions of glycosylated and secretory proteins were successfully achieved using this auto-inducible system. The efficient auto-inducible protein expression system based on nitrogen catabolite regulation might be useful for protein production in the laboratory and industrial applications.

## Results

### Design and construction of the NCR regulated NSAES

**Fig. 2:**
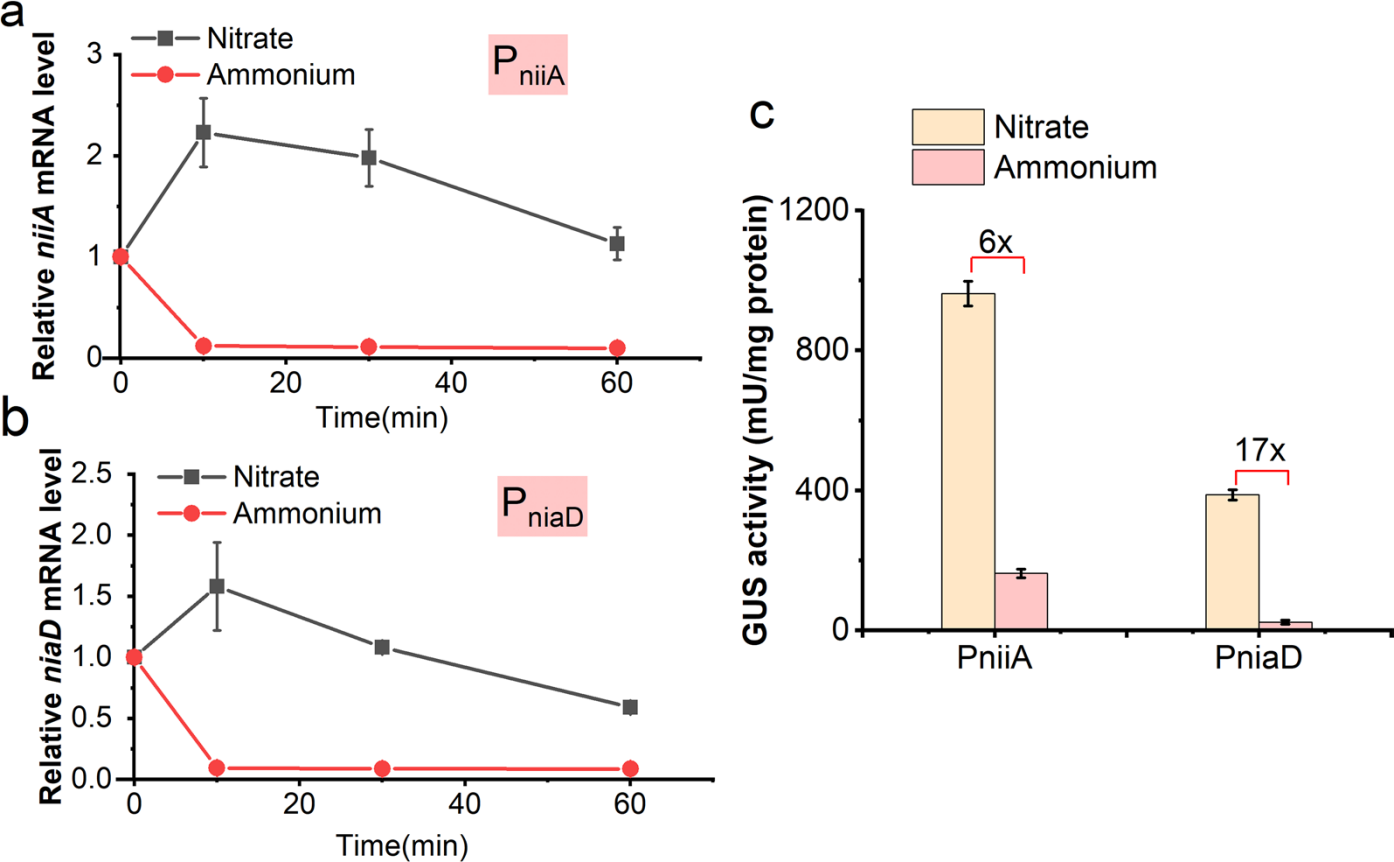
Characterization comparison of the P_niaD_ and P_niiA_. Transcription level of *niiA* gene (**a**) and *niaD* gene (**b**) in *A. nidulans*. (**c**). The activity of GUS controlled by P_niaD_ and P_niiA_. The time of mycelia transfer from 10 mM proline to 10 mM NO_3_^−^ or 10 mM NH_4_^+^ was 0 min

In recent years, a nitrate inducible expression system based on the bidirectional promoter P_niiA_/P_niaD_ has been applied in *A. nidulans* [25, 26]. *A. nidulans* utilizes NH_4_^+^ as a primary nitrogen source and NO_3_^−^ as a secondary nitrogen source via NCR. The key to this regulation is that the NiiA and NiaD expression is regulated by the bidirectional promoter P_niiA_/P_niaD_. Here, we utilized this distinct regulatory model to construct an NSAES to achieve auto-inducible expression of the gene of interest (GOI). The promoter P_niiA_/P_niaD_ consisted of two relatively independent promoters, P_niiA_ and P_niaD_, which were both repressed by NH_4_^+^ and induced by NO_3_^−^. Their transcriptional activities under NH_4_^+^ or NO_3_^−^ as a nitrogen source were first compared in the native genetic environment by quantifying the mRNA expression of *niiA* and *niaD* by quantitative reverse transcription-polymerase chain reaction (RT-qPCR). As shown in Fig. 2a and b, when the cells were introduced into the medium with 10 mM NH_4_^+^ as the sole nitrogen source, transcriptions of *niiA* and *niaD* were dramatically repressed in 10 min. In contrast, their transcription levels were significantly increased in a medium with 10 mM NO_3_^−^ as the sole nitrogen source. In addition, the transcriptional activity of P_niiA_ was stronger than that of P_niaD_.

To further evaluate the difference of induction ratio between P_niiA_ and P_niaD_, we constructed an artificial promoter-reporter assay system using glucuronidase (GUS) as the reporter. When cells were cultured with 10 mM NH_4_^+^, the expression level of GUS was extremely low, and the activity of P_niaD_ was significantly induced to approximately 17-fold with 10 mM NO_3_^−^ (Fig. 2c). Compared to P_niaD_, P_niiA_ expressed higher levels of GUS when induced with 10 mM NO_3_^−^. However, insufficient inhibition of 10 mM NH_4_^+^ led to high background activity of P_niiA_, with an induction ratio of six-fold. The findings indicate that P_niaD_, which is more effectively repressed by NH_4_^+^ and has a higher induction ratio, is the preferred promoter for the construction of NSAES.

### Characterization of the GOI expression profile in NSAES

**Fig. 3:**
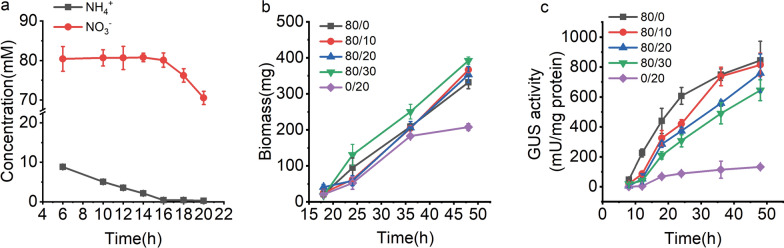
Effect of NO_3_^−^ and NH_4_^+^ concentrations on the auto-inducible expression system. **a** Confirmation of the utilization order of NO_3_^−^ and NH_4_^+^. **b** Biomass of A.n-PniaD-gusA under difference of NO_3_^−^/NH_4_^+^. **c** GUS activity under difference of NO_3_^−^/NH_4_^+^

Based on the regulation of NCR, it is conceivable that in the presence of NO_3_^−^ and NH_4_^+^, *A. nidulans* first consumes NH4^+^, which is also the basis for the construction of an auto-inducible expression system. To verify this, the order of NO_3_^−^ and NH_4_^+^ consumption was monitored under NO_3_^−^/NH_4_^+^ (80 mM/10 mM) culture condition. As shown in Fig. 3a, NH_4_^+^ was preferentially utilized during the early stages of fermentation. After NH_4_^+^ was consumed at < 2.1 mM, NO_3_^−^ was supplied as a secondary nitrogen source (Fig. 3a). This result demonstrates that the rigor of NCR regulation guarantees the sequential switching of nitrogen sources.

An appropriate nitrogen source ratio ensures low expression during the growth phase and high intensity during the expression phase. Therefore, we explored the biomass and GOI expression profiles of NSAES under different ratios of NO_3_^−^ and NH_4_^+^. Biomass increased steadily under the various cultivation conditions. The final biomass was higher in the medium with higher NO_3_^−^ and NH_4_^+^ concentrations. The findings confirm that NO_3_^−^ and NH_4_^+^ are efficient and compatible nitrogen sources (Fig. 3b). In contrast, GUS expression varied greatly in the different media (Fig. 3c). When NH_4_^+^ was used as the nitrogen source, GUS expression was extremely poor. With NO_3_^−^ as a nitrogen source, GUS expression was induced along with cell growth. GUS expression in a medium with a mixture of NO_3_^−^ and NH_4_^+^ clearly showed an auto-inducible characteristic; higher NH_4_^+^ concentration delayed GUS expression. Furthermore, with increased NH_4_^+^ concentration, GUS expression was gradually delayed. These findings show that the timing of GUS expression can be regulated by adjusting the amount of NH_4_^+^. Overall, NSAES demonstrated the ability to efficiently express heterologous proteins in an auto-inducible mode. This provided an auto-inducible expression model for developing other *Aspergillus* expression systems.

### Realization of high yield of host stress proteins by NSAES

The obvious advantage of the auto-inducible expression system is the ability to automatically switch between cell growth and production modes, which is of great importance in synthesizing heterologous proteins and metabolites under host stress. To validate the superiority of the production of host stress products, we established a toxic protein expression mode by employing PyrG orotidine 5′-phosphate decarboxylase. This is an essential enzyme for the de novo biosynthesis of pyrimidine. The absence of pyrimidine prevents the strain from surviving in media lacking uracil and uridine [27, 28]. PyrG catalyzes the transfer of fluorine from 5-fluoroorotic acid (5-FOA) to uracil to form 5-fluorouracil monophosphate, resulting in strong cytotoxicity. Therefore, PyrG was used to simulate the synthesis of host stress products.

**Fig. 4:**
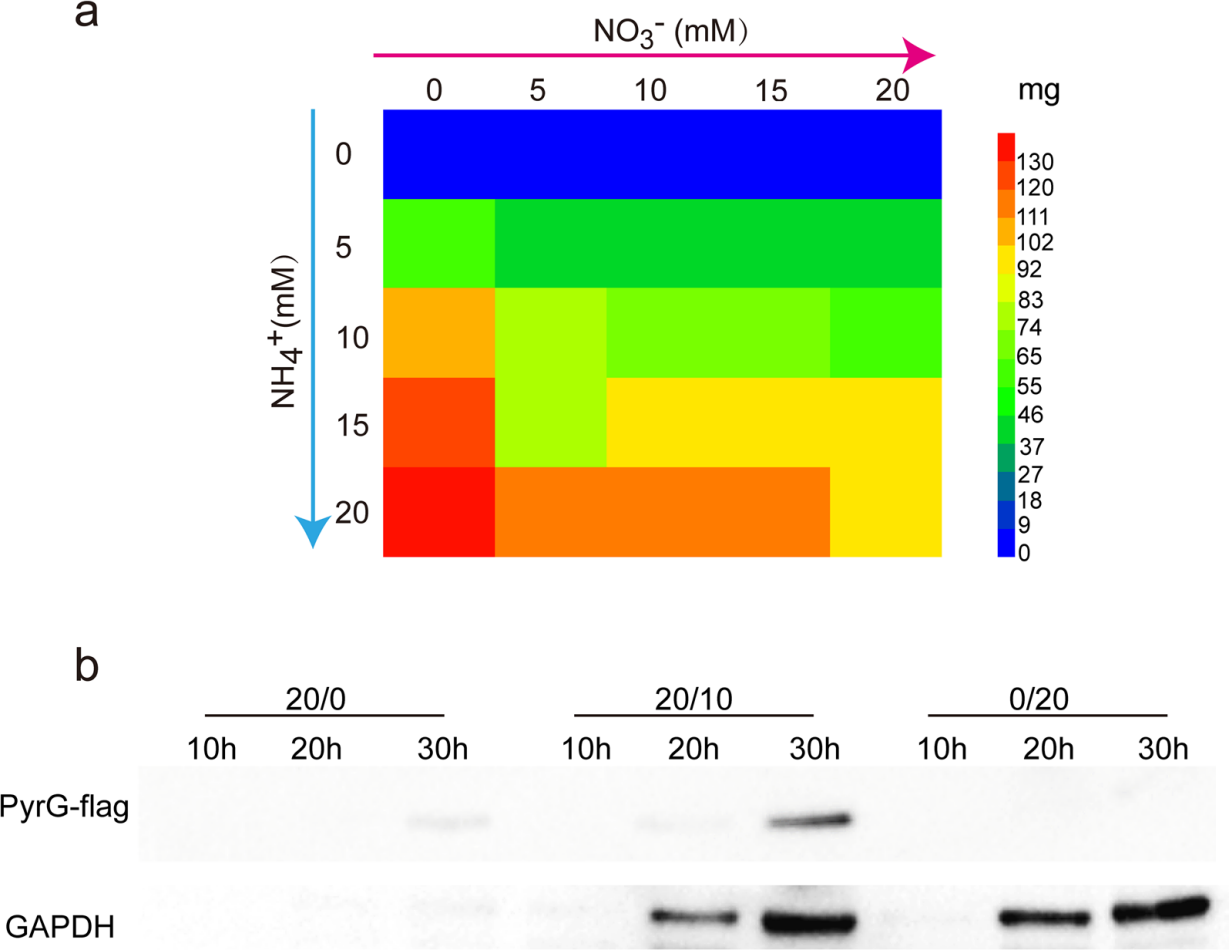
High yield of host stress protein by NSAES. **a** Biomass of A.n-PniaD-pyrG-flag under different NO_3_^−^/NH_4_^+^ concentrations in the presence of 5-FOA and uracil. **b** Western blot dection of PyrG and GAPDH. The A.n-PniaD-pyrG-flag strain was incubated under different NO_3_^−^/NH_4_^+^ concentration (20/0, 20/10, 0/20) in the presence of 5-FOA and uracil

Accordingly, the recombinant strain A.n-PniaD-pyrG-flag was constructed and cultured in media with various NO_3_^−^ and NH_4_^+^ concentrations, 0.3 mg/mL 5-FOA, and uracil. Biomass and PyrG expression levels were determined to evaluate the trade-off between cell growth and product synthesis. In media containing various concentrations of NO_3_^−^ but lacking NH_4_^+^, the biomass production was extremely low. When only NH_4_^+^ was used as the nitrogen source, the biomass production was substantially elevated with increasing NH_4_^+^ concentration owing to the suppression of PyrG expression (Fig. 4a). Cells cultured in NO_3_^−^/NH_4_^+^ mixed medium using the same total nitrogen source resulted in increased biomass with increasing NH_4_^+^ concentration. In contrast, biomass decreased with an increase in nitrate levels. Although the biomass increased as the total nitrogen source increased, more nitrogen sources did not increase the biomass since the biomass was 139.42 mg and 98.91 mg for 0/20 and 20/20 ratio of NO_3_^−^ to NH_4_^+^, respectively (Fig. 4a). All the biomass listed in Fig. 4a were provided in the Additional file 1: Table S3. These results showed that low biomass was caused by toxic protein expression and that the addition of NH_4_^+^ significantly reduced the stress of the toxic protein on the host, leading to higher biomass. The collective findings indicate that toxic protein expression can be achieved by appropriately adjusting the ratio of NO_3_^−^ and NH_4_^+^.

PyrG expression in different concentrations of NO_3_^−^ and NH_4_^+^ were detected by western blotting analysis. As shown in Fig. 4b, the constitutive expression of toxic protein under NO_3_^−^ as a nitrogen source severely inhibited cell growth; in turn, the final protein yield was poor owing to insufficient biomass production. This negligible increase in biomass with NH_4_^+^ as a nitrogen source did not contribute to the final product accumulation. However, in the NO_3_^−^/NH_4_^+^ mixed medium, the expression of PyrG was significantly improved, especially at 30 h. The biomass was similar to NH_4_^+^ as a nitrogen source, but the specific band of PyrG was more prominent. These results suggest that after switching the nitrogen source from NH_4_^+^ to NO_3_^−^, PyrG production was significantly improved, benefiting from the higher biomass. The simulation based on PyrG demonstrated that NSAES were capable of high-level synthesis of host stress products, such as toxic proteins and metabolites, through an automatic switch from the growth mode to the production mode.

### Overexpression of high molecular weight glycosylated proteins by NSAES

Efficient expression of high molecular weight proteins remains a major challenge for various expression systems. In addition, glycosylation modification in filamentous fungi is closest to that occurring in mammals. In contrast, bacterial expression systems do not feature glycosylation modifications, whereas yeasts are prone to excessive glycosylation [29]. Catalase (CatB) is a native tetramer glycosylated protein containing four monomers with a molecular mass of 90 kDa [30]. In the present study, CatB was used to verify the advantage of NSAES for the overexpression of high molecular weight glycosylated proteins.

**Fig. 5:**
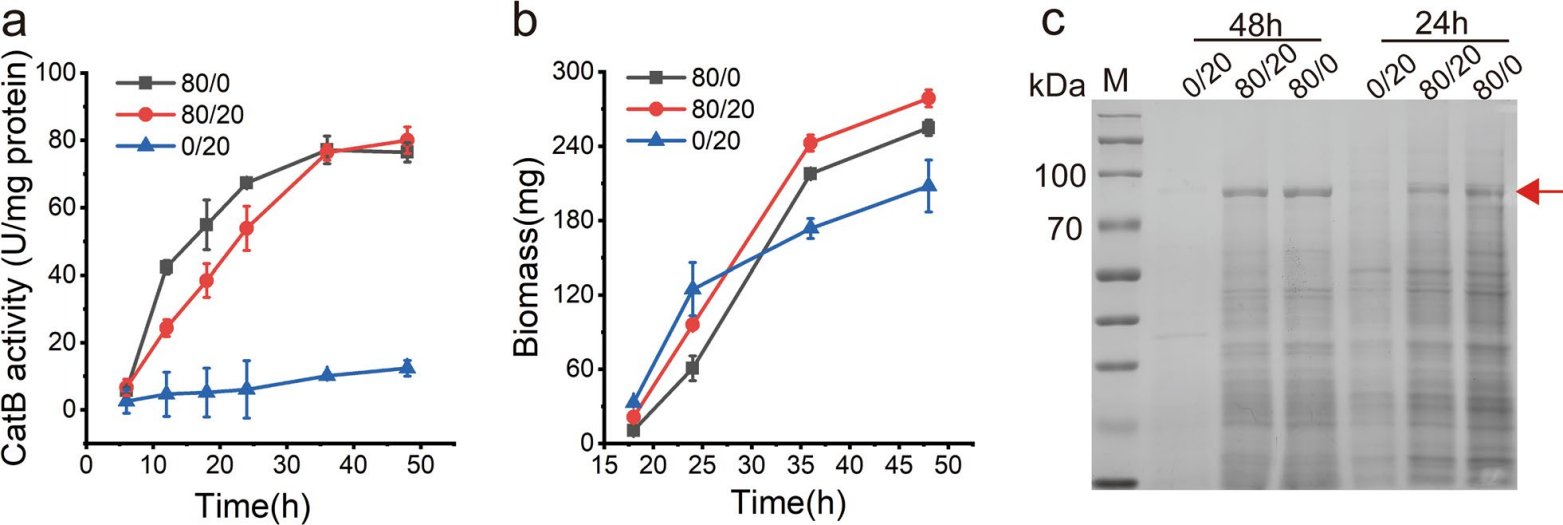
Overexpression of CatB in NSAES. **a** CatB activity in difference of NO_3_^−^/NH_4_^+^ concentrations. **b** Biomass of A.n-PniaD-CatB in difference of NO_3_^−^/NH_4_^+^ concentrations. **c** SDS-PAGE analysis of CatB. The red arrow points to the CatB

CatB activity was low under NO_3_^−^ and NH_4_^+^ conditions in the wild-type strain ABPUN, but the recombinant strain A.n-PniaD-CatB demonstrated significant activity under only NO_3_^−^ (Additional file 1: Fig. S1a). SDS-PAGE revealed that CatB was highly efficient expression in the recombinant strain (Additional file 1: Fig. S1b). The recombinant strain was cultured in a medium containing 20 mM NH_4_^+^ and 80 mM NO_3_^−^ for the auto-inducible expression of CatB. Constitutive expression (80 mM NO_3_^−^) and repressed expression (20 mM NH_4_^+^) were also set. Biomass and enzymatic activity were monitored at appropriate time intervals. Consistent with expectations, CatB could not be induced in the absence of NO_3_^−^. The finding demonstrated the low leaky expression of NSAES (Fig. 5a). In the medium supplemented with only NO_3_^−^, the expression of CatB was accompanied by cell growth and eventually reached high levels (Fig. 5a). In the auto-inducible expression mode, despite delayed induction, the expression level of CatB increased rapidly after the onset of induction and eventually was slightly higher than in the case of constitutive expression (Fig. 5a). Moreover, similar to CatB expression, culture in the auto-inducible mode also increased the biomass (Fig. 5b). The high level of CatB was verified by SDS-PAGE (Fig. 5c). These collective results indicate the capability of NSAES for the highly efficient expression of high molecular weight glycosylated proteins, wherein the auto-inducible mode exhibited further improvement.

### Secretory expression of xylanase by NSAES

**Fig. 6:**
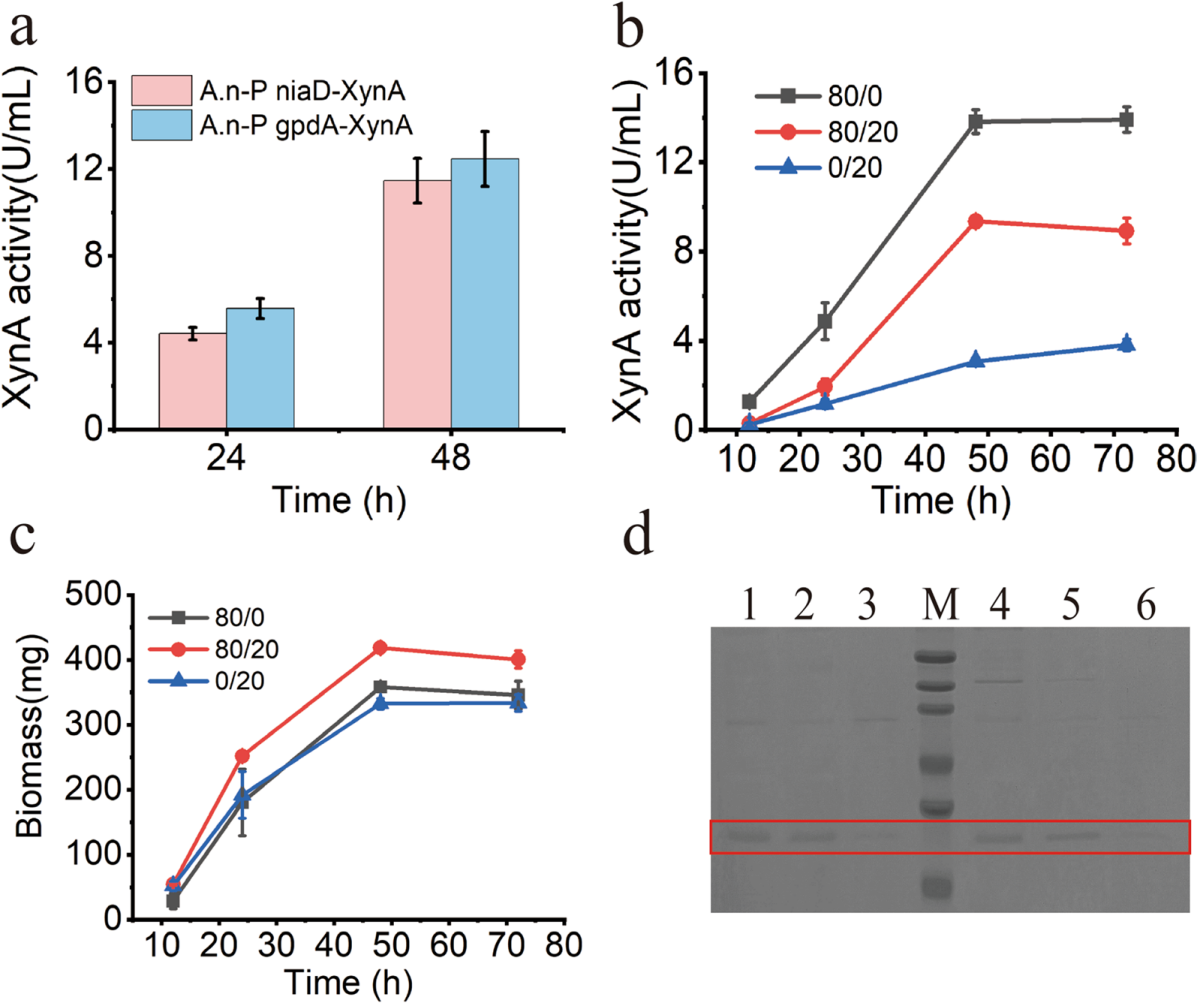
Secretory expression of xylanase by NSAES. **a** Comparison of XynA in NSAES and a strong constitutive expression system in the 80 mM NO_3_^−^ condition. **b** XynA activity in difference of NO_3_^−^/NH_4_^+^ concentrations. **c** Biomass of A.n-PniaD-XynA in difference of NO_3_^−^/NH_4_^+^ concentrations. **d** SDS-PAGE analysis of expressed XynA. 1, 2, 3, XynA expression at 48 h in NO_3_^−^/NH_4_^+^ concentration of 80/0, 80/20 and 0/20; 4, 5, 6, XynA expression at 72 h in NO_3_^−^/NH_4_^+^ concentration of 80/0, 80/20 and 0/20; Mark:70, 50, 40, 35, 25 and 15 kDa

The main advantage of using filamentous fungi as an expression host is their excellent secretion efficiency [31, 32]. Therefore, the ability of the NSAES platform to induce the expression of secretory proteins was evaluated. Xylanase is an industrial enzyme that plays an important role in degrading cellulose and hemicellulose. Xylanase activity was undetected in the wild-type strain under the culture conditions used in this study (data not shown). In this study, we constructed a recombinant strain expressing the endogenous xylanase XynA (A.n-PniaD-XynA) using NSAES. First, we compared the XynA expression controlled by the strong constitutive promoter P_gpdA_ and the NO_3_^−^ inducible promoter P_niaD_. The activity of P_niaD_ was strong (Fig. 6a). Next, A.n-PniaD-XynA was cultured in various ratios of NO_3_^−^/NH_4_^+^ (80 mM/0 mM, 80 mM/20 mM, and 0 mM/20 mM). The activities of XynA were low throughout the cultivation process in a medium containing only NH_4_^+^ (Fig. 6b). Culturing at 80 mM/0 mM and 80 mM/20 mM NO_3_^−^/NH_4_^+^ led to high XynA activity. Notably, the auto-inducible mode did not show an advantage over the constitutive mode in XynA expression, which was similar to the expression of GUS. However, the biomass of 80/0, 80/20, and 0/20 was 358.05 ± 3.18 mg, 418.60 ± 2.82 mg, and 332.50 ± 8.06 mg, respectively. The biomass cultured in the auto-inducible mode was relatively higher. (Fig. 6c). The high expression of XynA was verified by SDS-PAGE (Fig. 6d). The collective findings indicate the suitability of NSAES for the expression of secreted proteins.

## Discussion

CCR regulation is the most common prototype for constructing inducible expression systems in various organisms. The diverse regulators, including transcription factors, promoters, and riboswitches, have been deeply mined and adaptively engineered [33–35]. Moreover, owing to the rigorous sequential utilization of different carbon sources by organisms, the development of auto-inducible expression systems is possible simply by mixing different carbon sources. For instance, the classic lac operon has been employed to construct a lactose/IPTG-inducible expression system. On this basis, GOI can be expressed in an auto-inducible manner in media containing glucose and lactose [3]. However, in some cases, the use of expression systems induced by carbon sources has been limited. Regulation of CCR is very complicated, involving the phosphotransferase system, ATP regeneration, and organic acid synthesis [36, 37]. Changes in these regulatory networks have a significant effect on cellular metabolism. However, the carbon source-inducible expression system is not suitable for synthesizing metabolites using carbohydrates as the backbone molecules, such as the production of inositol from glucose and xylitol from xylose [38, 39].

In comparison, the mining and application of regulators in NCR regulatory networks have not been as intensively explored. In this study, we developed an NSAES based on NCR. The promoter P_niaD_, whose transcription is inhibited by NH_4_^+^ and activated by NO_3_^−^, was used in the NSAES because of its high activity and induction ratio (Fig. 2). GUS was used as the reporter for the characterization of NSAES. In a medium containing NH_4_^+^ and NO_3_^−^ complex medium, NSAES could express GOI in an auto-inducible manner. For the expression of all proteins tested in this study, including GUS, CatB and XynA, when NSAES was applied, the timing of protein expression was apparently delayed depending on the concentrations of NH_4_^+^. For instance, under the typical condition (80/20 of NO_3_^−^/NH_4_^+^), the GOI expression is clearly divided into two phases, the inhibition in the early stage and induction in the latter stage. GOI overexpression has the detrimental effect of reducing the substances and energy supplied to cell growth. We also found that cell growth under auto-inducible mode (80/20) was apparently faster than that under the constitutive mode (80/0) (Figs. 5b and 6b). These results showed the switching between growth mode and production mode of cells. Notably, delayed GUS induction resulted in a final lower expression level, despite the same concentration of NO_3_^−^ (Fig. 3). A similar phenomenon was observed when XynA was expressed (Fig. 6). These results indicate that constitutive expression is more advantageous than that induced during growth to produce proteins without apparent host stress.

The production of host toxic proteins poses a challenge. Delaying the synthesis of toxic products during fermentation is an effective strategy to solve this problem. In this study, NSAES efficiently decoupled cell growth and production through automatic switching of nitrogen sources (Fig. 3). Based on this, PyrG was used to establish a model to simulate the synthesis of host toxic products. In this model, NSAES reduced the stress on the host caused by the synthesis of toxic products. High yields of biomass and host toxic products can be obtained simultaneously using NSAES. This result conclusively demonstrates the advantages of NSAES in regulating the synthesis of various products and the broad application prospects of this system in protein expression, metabolic engineering, and synthetic biology.

Glycosylation can play a vital role in the activity and half-life of proteins, including enzymes and antibodies. At present, the main therapeutic production hosts are mammalian cells because their protein modification system is similar to that of humans. However, the genetic manipulation of mammalian cells is inconvenient, and their culture is expensive and lengthy. *A. nidulans* possesses a protein modification system similar to that of humans. Thus, it is an ideal alternative host for mammalian cells. Recently, several studies described the expression of therapeutic proteins in filamentous fungi, such as the production of whole humanized immunoglobulin G1-kappa antibodies in *A. niger* at titers of 0.2–0.9 g/L and the expression of human interferon beta (HuIFNβ) in *A. unguis* [40, 41]. Moreover, the *A. nidulans* glycosylation system can be made closer to humans by glycoengineering [42]. Glycosylated protein expression in NSAES was demonstrated by the highly efficient expression of CatB. The finding indicates that NSAES could be an excellent platform for producing therapeutic proteins.

Compared with other filamentous fungi, *A. nidulans* is rarely used in the scaled-up production of industrial enzymes because of their low expression capacity. *Trichoderma reesei* is the main strain for cellulase production because of its excellent secretory ability [43, 44]. However, the fermentation process of *T. reesei* usually takes up to a week. *A. nidulans* fermentation occurs in under three days and is, therefore, a candidate strain for the scaled-up production of industrial enzymes. Recently, several strategies have been developed to improve protein production by *A. nidulans*. For instance, when the Hsp40 co-chaperone *ydjA* gene was knocked out, xylanase XlnE production increased by 171% [45]. *A. nidulans* production of cellulase production was improved in the Δ*pkaA* (protein kinase A) strain [46]. In this study, the high strength of P_niaD_ was verified by the expression of CatB and XynA (Figs. 5 and 6). Therefore, NSAES is expected to be an ideal platform for scale-up production of industrial enzymes in the future.

## Conclusions

In this study, we constructed and characterized an NSAES based on NCR. The NSAES can trade-off the production of host stress proteins and growth, effectively expresses high molecular weight glycoprotein, and is expected to be developed as a candidate strain for cellulase production. This efficient auto-inducible protein expression system based on nitrogen catabolite regulation will be valuable for protein production in the laboratory and industrial applications.

## Methods

### Strains and culture conditions

*Escherichia coli* JM109 was used as host for DNA manipulation. All transformants were grown on LB supplemented with 100 µg/mL ampicillin. *A. nidulans* ABPUN was used as parental strain. The glucose minimal medium (GMM) was used for the culture of *A. nidulans*. The ABPUN was culture by GMM with 0.5 g/L arginine, 0.5 g/L uracil, 0.6 g/L uridine, 0.4 g/L biotin B and 0.4 g/L pyridoxine and adjust pH 6.5 at 37 ℃. All *A. nidulans* transformants were cultured by GMM with the 0.5 g/L uracil, 0.6 g/L uridine, 0.4 g/L biotin B and 0.4 g/L pyridoxine and adjust pH 6.5 at 37 ℃.

### Plasmid construction

The seamless cloning method was used for plasmid construction. The high-fidelity PrimeSTAR^®^ Max DNA Polymerase (Takara bio Inc., Japan) was used for gene and vector fragments amplification. Then, the PCR products were treated with DpnI to remove the template and cleaned up. Finally, the purified fragments were seamlessly joined using Hieff Clone^®^ Plus One Step Cloning Kit (Yeasen, China) ligation and the reaction product was directly transformed into JM109 competent cells. Plasmids were introduced into *A. nidulans* ABPUN using obtain recombinant *A. nidulans* strains (Additional file 1: Fig. S2). All plasmids and strains are listed in Additional file 1: Table S1. All primers are listed in Additional file 1: Table S2.

### RT-qPCR assay

ABPUN conidia were collected with 0.85% (m/v) sodium chloride containing 0.2‰ (v/v) Tween 80. For prepare the total RNA, 1 × 10^7^ conidia of ABPUN strain were cultured at 37 ℃, 220 rpm in GMM with 10 mM proline for pre-cultured. Then the mycelia were transfer to fresh medium with 10 mM NO_3_^−^ or 10 mM NH_4_^+^ and the mycelia were harvested at 0 min, 10 min, 30 min, 60 min, respectively. The time of mycelia transfer from 10 mM proline to 10 mM NO_3_^−^ or 10 mM NH_4_^+^ was 0 min. Total RNA extraction using RNApure Plant kit (CWbiotect, China). The reverse transcription was performed with the HiFiScript gDNA Removal RT MasterMix Kit (CWbiotect, China). Then, the real time PCR was carried with the MagicSYBR Mixture Kit (CWbiotect, China), and the CFX96 Touch™ Real-Time PCR Detection System (Bio-Rad Laboratories, Inc., USA). The real-time PCR program was as follows: 95 ℃ for 30 s; then 40 cycles of 95 ℃ for 5 s and 60 ℃ for 30 s; then 95 ℃ for 10 s; then 60 ℃ for 30 s and increasing to 95 ℃ to test the melt curve. Quantitative PCR experiment data analysis was performed by the 2^−△△Ct^ method. Relative mRNA was normalized to the reference gene, actinA. All quantitative PCR primers are shown in Additional file 1: Table S2.

### Determination of the concentration NO_3_^−^ and NH_4_^+^

To detect the NO_3_^−^ and NH_4_^+^, 1 × 10^6^ conidia of A.n-PniaD-gusA strain was cultured at 37 ℃, 220 rpm in GMM with the NO_3_^−^/ NH_4_^+^ (80 mM/10 mM) and the fermentation supernatants were sampled at 6 h, 10 h, 12 h, 14 h, 16 h, 18 h, 20 h for NO_3_^−^ and NH_4_^+^ determination. The NH_4_^+^ concentration was measured using the Ammonia Assay Kit (sigma, USA). The NO_3_^−^ was analyzed on High Performance Ion Chromatography (HPIC) equipped with an iopnac AS11 analytical column. A 30 mM NaOH solution was used as eluent at a flow rate of 1.5 mL/min for 15 min at 30 °C.

### Enzymatic activity assay

The mycelia were harvested and ground to extract crude protein. Preparation of cell-free extracts was performed as described previously [47]. Intracellular protein extract buffer was composed of 50 mM sodium phosphate buffer pH 7.0, 10 mM β-mercaptoethanol, 10 mM Na_2_EDTA, and 0.1% (v/v) Triton X-100. Bradford method was used to determine the protein concentration. The activity of GUS was measured using 4-methylumbelliferyl-b-glucuronide (4-MUG) as substrate, and quantified by the fluorescence intensity of the product 4-methylumbelliferone (4-MU) [48]. The fluorescence intensity of 4-MU was detected with microplate reader with the excitation wavelength of 365 nm and the emission wavelength of 455 nm. One unit (mU) was defined as the amount of enzyme required to produce 1 pM 4-MU in 1 minute under 2 mM 4-MUG at 37 ℃.

The CatB activity was measured using H_2_O_2_ as the substrate [49]. H_2_O_2_ was detected with microplate reader with a wavelength of 240 nm. The enzyme unit was defined as the amount of enzyme required to decrease the OD by 0.01 in one second under the 0.1% H_2_O_2_ at 30 ℃.

Xylanase activity was using dinitrosalicylic acid (DNS) method against xylan. Crude enzyme samples were centrifuged at 12,000 rpm (4 °C, 10 min), and the supernatants were transferred into a new centrifuge tube and placed on ice until used in enzyme assays. A 40 µL volume of xylan (1%) for xylanase activity was added to 60 µL crude enzyme solution (diluted to the appropriate concentration using HAC-NaAC buffer, pH 5.0). The resulting reaction mixtures were mixed well and incubated at 52 °C for 30 min Then, a 300 µL volume of DNS was added to terminate the reactions. The reaction mixtures were subsequently placed in boiling water for 5 min. Distilled water was added to maintain a constant volume of 400 µL as the liquid cooled. The optical densities (ODs) of the reaction solutions were determined using a microplate reader at a wavelength of 540 nm. Enzyme activity unit for xylanase was defined as the amount of enzyme required to produce 1 µmol of xylose per minute under experimental conditions.

### SDS-PAGE and western blot

For SDS-PAGE, 20 µL of protein sample was mixed with 5 µL of 5×Loading buffer and boiled for 10 min before loading. After electrophoresis, the gel was retained using Coomassie Brilliant Blue R-250. For western blot, all the mycelia from each condition were harvested and ground to extract crude protein with equal volumes of buffer, then the 10 µL protein sample for GAPDH, and the 3 µL 10 times diluted protein solution for PyrG-flag were loaded on 12% SDS-PAGE and then transferred to PVDF membranes. Non-specific antigen binding was blocked with TBS-T (20mM Tris, 137mM NaCl and 0.1% Tween-20) with 5%(m/V) skimmed milk powder for 1 h. Then, membranes were washed 10 min with TBS (20mM Tris, 137mM NaCl and 20% methanol). Membranes were incubated with anti-flag-tag (TransGen Biotech, China) or anti-GAPDH (TransGen Biotech, China) primary antibodies for 2 h. Membranes were washed 3 10 min washes with TBS and anti-flag-tag/anti-GAPDH membranes were further incubated with anti-mouse antibody/anti-rabbit antibody for 2 h followed by 3 10 min washes in TBS. The band of proteins were visualized with boyoECL Plus kit (Solarbio, China) and imaged using the ChemiDoc MP Imaging System (Bio-Rad).

### Biomass determination

The fresh mycelia were harvested and placed in the oven to dry at 100 ℃ to constant weight.

## Supplementary Information

**Additional file 1:** **Figure S1.** Overexpression of CatB in NSAES **a** CatB activity of ABPUN and A.n-PniaD-CatB. **b** SDS-PAGE of CatB at 24 h. **Figure S2. **Construction of recombinant *Aspergillusnidulans *strains. Strategy for homologoustransformation to insert the expression plasmid. **FigureS3. **PniiA/PniaD bidirectionalpromoter sequence and annotation. Four NirA binding sites (red) and ten AreA binding sites (blue). **Table S1.** *Aspergillus nidulans* strains usedin this study. **Table S2.** Primers used in this study. **Table S3.** Biomass of strainA.n-P_niaD_-pyrG-flag cultured in difference NO_3_^−^/NH_4_^+^ratio.

## Data Availability

The datasets used during the current study are available from the corresponding author on reasonable request.
