# Supplementary material for: Development of an auto-inducible expression system by nitrogen sources switching based on the nitrogen catabolite repression regulation
Source: Microb Cell Fact. 2022 Apr 28;21:73. doi: 10.1186/s12934-022-01794-5 (PMC9047365; doi:10.1186/s12934-022-01794-5)
Supplement: Supplementary file 1 — Additional file 1: Figure S1. Overexpression of CatB in NSAES a CatB activity of ABPUN and A.n-PniaD-CatB. b SDS-PAGE of CatB at 24 h. Figure S2. Construction of recombinant Aspergillusnidulans strains. Strategy for homologoustransformation to insert the expression plasmid. FigureS3. PniiA/PniaD bidirectionalpromoter sequence and annotation. Four NirA binding sites (red) and ten AreA binding sites (blue). Table S1. Aspergillus nidulans strains usedin this study. Table S2. Primers used in this study. Table S3. Biomass of strainA.n-PniaD-pyrG-flag cultured in difference NO3−/NH4+ratio. [file 12934_2022_1794_MOESM1_ESM.docx]

**Supplementary material**

**Development of an auto-inducible expression system by nitrogen sources switching based on the nitrogen catabolite repression regulation**

Qin Yan^1#^, Laichuang Han ^1#^, Xinyue Liu^1^, Cuiping You^1^, Shengmin Zhou^2*^, Zhemin Zhou^1*^

^1^ Key Laboratory of Industrial Biotechnology (Ministry of Education), School of Biotechnology, Jiangnan University, Wuxi, Jiangsu, China

^2^ State Key Laboratory of Bioreactor Engineering, School of Biotechnology, East China University of Science and Technology, Shanghai, China

*^#^* These authors contributed equally to this work.

*corresponding authors：

Shengmin Zhou,

State Key Laboratory of Bioreactor Engineering, School of Biotechnology, East China University of Science and Technology, Shanghai, China

E-mail: zhoushengmin@ecust.edu.cn

Zhemin Zhou,

School of Biotechnology, Jiangnan University

Wuxi, Jiangsu, 214122, China

E-mail: [zhmzhou@jiangnan.edu.cn](mailto:zhmzhou@jiangnan.edu.cn)


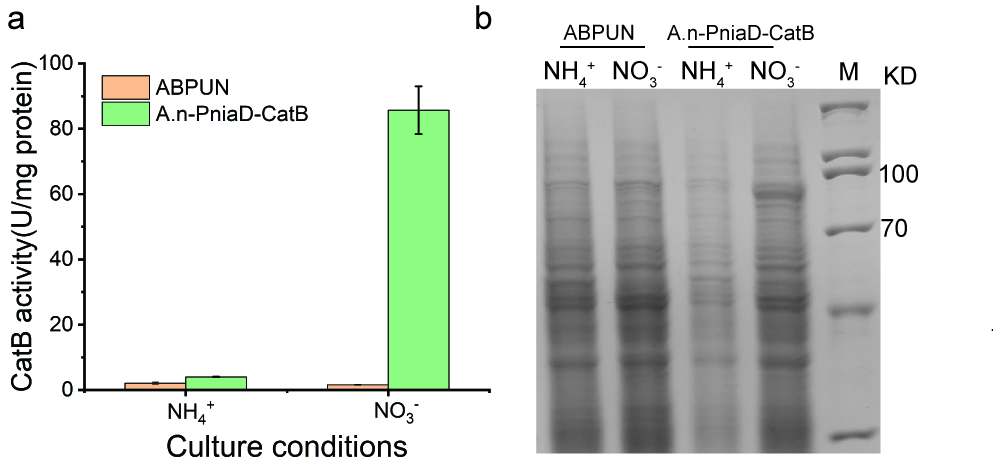


**Fig. S1 Overexpression of CatB in NSAES** a) CatB activity of ABPUN and A.n-PniaD-CatB. b) SDS-PAGE of CatB at 24 h.


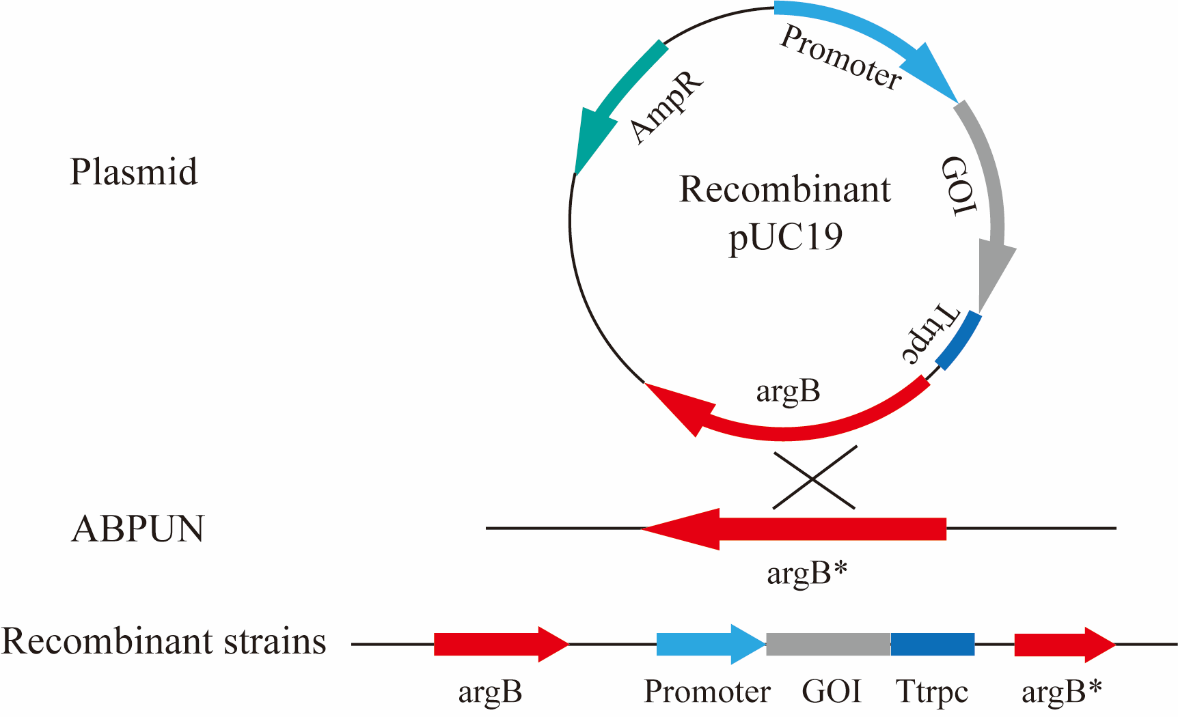


**Fig. S2 Construction of recombinant *Aspergillus nidulans* strains.**

Strategy for homologous transformation to insert the expression plasmid.


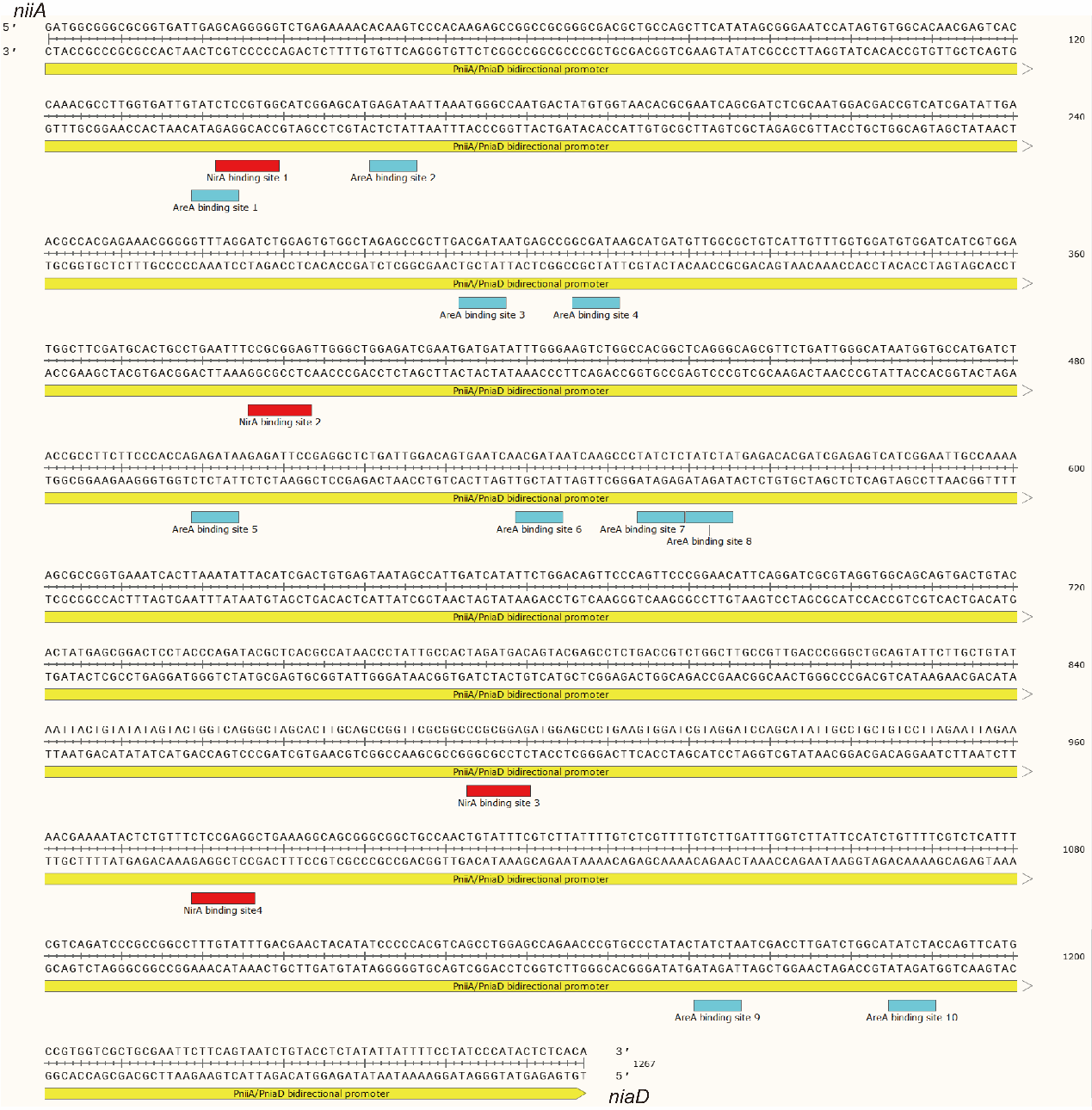


**Fig. S3** PniiA/PniaD bidirectional promoter sequence and annotation**.**

**Four NirA binding sites (red) and ten AreA binding sites (blue)**

**Tab. S1 *Aspergillus nidulans* strains used in this study.**

| Strain | Genotype | Source |
| --- | --- | --- |
| ABPUN | *biA1*; *pyrG89*; *yA*; *argB2*; *pyroA4* | Lab stock |
| A.n-PniaD-gusA | biA1; pyrG89; yA; pyroA4;  ΔargB::PniaD gusA TtrpC argB2 pUC19 | This work |
| A.n-PniiA-gusA | biA1; pyrG89; yA; pyroA4;  ΔargB::PniaD gusA TtrpC argB2 pUC19 | This work |
| A.n- PniaD-PyrG-flag | biA1; pyrG89; yA; pyroA4;  ΔargB::PniaD pyrG TtrpC argB2 pUC19 | This work |
| A.n- PniaD-CatB | biA1; pyrG89; yA; pyroA4;  ΔargB::PniaD CatB TtrpC argB2 pUC19 | This work |
| A.n- PniaD-XynA | biA1; pyrG89; yA; pyroA4;  ΔargB::PniaD XlnA TtrpC argB2 pUC19 | This work |
| A.n- PgpdA-XynA | biA1; pyrG89; yA; pyroA4;  ΔargB::PgpdA XlnA TtrpC argB2 pUC19 | This work |
| Plasmids |  |  |
| pUC-PniaD-gusA | PniaD gusA TtrpC; argB; Amp | This work |
| pUC-PniiA-gusA | PniiA gusA TtrpC; argB; Amp | This work |
| pUC-PniaD-PyrG | PniaD PyrG TtrpC; argB; Amp | This work |
| pUC-PniaD-CatB | PniaD CatB TtrpC; argB; Amp | This work |
| pUC-PniaD-XynA | PniaD XynA TtrpC; argB; Amp | This work |
| pUC-PgpdA-XynA | PgpdA XynA TtrpC; argB; Amp | This work |

**Tab. S2 Primers used in this study**

| Primer name | sequence |
| --- | --- |
| Pn-gus-F | TCCTATCCCATACTCTCACAATGTTACGTCCTGTAGAAACCC |
| gus-R-Trpc | ATGGAGCTATTAAATCACTATCATTGTTTGCCTCCCTGCTGCG |
| PniaD-F/PniiA-R | GATGGCGGGCGCGGTGATT |
| PniaD-R/PniiA-F | TGTGAGAGTATGGGATAGGA |
| TrpC-F | TAGTGATTTAATAGCTCCATG |
| Pn-catB-F | TCCTATCCCATACTCTCACAATGCGAGCTCTCGGCCTGGT |
| catB-R-Ttrpc | ATGGAGCTATTAAATCACTACTATTCATCCGAGTCCAGGGC |
| Pn-pyrG-F | TCCTATCCCATACTCTCACAATGTCTTCGAAGTCCCACC |
| pyrg-R-flag | CACTTGTCATCGTCGTCCTTGTAATCAAGTCCAACTCTTTTCTCGT |
| flag-Tt-F | AAGGACGACGATGACAAGTGATGATAGTGATTTAATAGCTCCATG |
| Pn-XynA-F | TCCTATCCCATACTCTCACAATGGTCTCCTTCAAATCTCTCC |
| XynA-R-Tt | ATGGAGCTATTAAATCACTACTAGTAAACAGTAATAGAAGCCG |
| qPCR primer |  |
| q-niiA-F | GTATCATTGCTCCTGGTATTG |
| q-niiA-R | AATCTCTTCGGTTCCTTGT |
| q-niaD-F | GGATGCTTCTGAGGAGTT |
| q-niaD-R | GTGCCGATATGGTAGTCT |
| q-actA-F | GGTCTGGAAAGCGGTGGTATC |
| q-actA-R | CCAGGGTACATGGTGGTACC |

**Tab. S3 Biomass of strain A.n-P_niaD_-pyrG-flag cultured in difference NO_3_^-^/NH_4_^+^ ratio**

| NO_3_ (mM)  NH_4_^+^(mM) | 0 | 5 | 10 | 15 | 20 |
| --- | --- | --- | --- | --- | --- |
| 0 | 0 | 6.0 | 5.2 | 7.5 | 5.2 |
| 5 | 57.6 | 41.7 | 39.1 | 45.5 | 42.5 |
| 10 | 103.8 | 81.6 | 68.2 | 70.4 | 57.9 |
| 15 | 129.1 | 80.9 | 93.9 | 97.0 | 94.9 |
| 20 | 139.4 | 118.6 | 112.2 | 115.0 | 98.9 |
